# A human pluripotent stem cell-derived in vitro model of the blood–brain barrier in cerebral malaria

**DOI:** 10.1186/s12987-024-00541-9

**Published:** 2024-05-01

**Authors:** Adnan Gopinadhan, Jason M. Hughes, Andrea L. Conroy, Chandy C. John, Scott G. Canfield, Dibyadyuti Datta

**Affiliations:** 1 Ryan White Center for Pediatric Infectious Disease and Global Health, Indiana University School of Medicine, R4-402D 1044 W. Walnut St., Indianapolis, IN 46202 USA; 2 Department of Microbiology and Immunology, Indiana University School of Medicine, Indianapolis, IN 46202 USA; 3 Department of Anatomy, Cell Biology, and Physiology, Indiana University School of Medicine, 620 Chestnut Street, Terre Haute, IN 47809 USA; 4 Stark Neurosciences Research Institute, Indiana University School of Medicine, Indianapolis, IN 46202 USA

**Keywords:** Blood–brain barrier, Cerebral malaria, *Plasmodium falciparum-*infected red blood cells, Human induced pluripotent stem cell-derived brain microvascular endothelial cells

## Abstract

**Background:**

Blood–brain barrier (BBB) disruption is a central feature of cerebral malaria (CM), a severe complication of *Plasmodium falciparum* (*Pf*) infections. In CM, sequestration of *Pf*-infected red blood cells (*Pf*-iRBCs) to brain endothelial cells combined with inflammation, hemolysis, microvasculature obstruction and endothelial dysfunction mediates BBB disruption, resulting in severe neurologic symptoms including coma and seizures, potentially leading to death or long-term sequelae. In vitro models have advanced our knowledge of CM-mediated BBB disruption, but their physiological relevance remains uncertain. Using human induced pluripotent stem cell-derived brain microvascular endothelial cells (hiPSC-BMECs), we aimed to develop a novel in vitro model of the BBB in CM, exhibiting enhanced barrier properties.

**Methods:**

hiPSC-BMECs were co-cultured with HB3var03 strain *Pf-*iRBCs up to 9 h. Barrier integrity was measured using transendothelial electrical resistance (TEER) and sodium fluorescein permeability assays. Localization and expression of tight junction (TJ) proteins (occludin, zonula occludens-1, claudin-5), cellular adhesion molecules (ICAM-1, VCAM-1), and endothelial surface markers (EPCR) were determined using immunofluorescence imaging (IF) and western blotting (WB). Expression of angiogenic and cell stress markers were measured using multiplex proteome profiler arrays.

**Results:**

After 6-h of co-culture with *Pf-*iRBCs, hiPSC-BMECs showed reduced TEER and increased sodium fluorescein permeability compared to co-culture with uninfected RBCs, indicative of a leaky barrier. We observed disruptions in localization of occludin, zonula occludens-1, and claudin-5 by IF, but no change in protein expression by WB in *Pf-*iRBC co-cultures. Expression of ICAM-1 and VCAM-1 but not EPCR was elevated in hiPSC-BMECs with *Pf-*iRBC co-culture compared to uninfected RBC co-culture. In addition, there was an increase in expression of angiogenin, platelet factor-4, and phospho-heat shock protein-27 in the *Pf-*iRBCs co-culture compared to uninfected RBC co-culture.

**Conclusion:**

These findings demonstrate the validity of our hiPSC-BMECs based model of the BBB, that displays enhanced barrier integrity and appropriate TJ protein localization. In the hiPSC-BMEC co-culture with *Pf*-iRBCs, reduced TEER, increased paracellular permeability, changes in TJ protein localization, increase in expression of adhesion molecules, and markers of angiogenesis and cellular stress all point towards a novel model with enhanced barrier properties, suitable for investigating pathogenic mechanisms underlying BBB disruption in CM.

**Supplementary Information:**

The online version contains supplementary material available at 10.1186/s12987-024-00541-9.

## Background

Cerebral malaria (CM) presents clinically as an unarousable coma and remains a significant driver of childhood mortality and neurodisability. The pathogenesis of CM involves sequestration of *P. falciparum-*infected red blood cells (*Pf-*iRBCs) to the brain endothelium, widespread inflammation, hemolysis and cellular damage, microvascular obstruction, accumulation of toxic parasite by-products resulting from parasites bursting out of RBCs, and parasite-mediated disruption of endothelial and tight junction (TJ) barrier integrity of the brain’s microvasculature [1–6]. Markers of neuronal injury, such as tau, are elevated in the cerebrospinal fluid in CM, and can be detected at higher levels in blood-circulation compared to healthy controls, suggesting possible transport across a damaged BBB [7, 8]. What remains unknown are the specific mechanisms by which *Pf*-iRBCs confined within the vascular space disrupt barrier integrity and brain function without crossing the blood–brain barrier (BBB) in CM. A dearth of biologically and physiologically relevant experimental models of the BBB to study the pathogenic pathways underlying brain injury in CM is a key contributor to this gap in knowledge.

In post-mortem studies, brain biopsies from patients have identified damage to the BBB resulting from sequestration of *Pf-*iRBCs in the brain microvasculature, including decreased expression of TJ proteins, occludin and zonula occludens-1 (ZO-1), expressed on brain microvascular endothelial cells (BMECs) [3, 9], and evidence of serum proteins such as albumin leaking across the BBB [1, 10]. Brain imaging studies have enhanced our understanding of the role of brain swelling and herniation as a contributor to mortality in children and adults with CM [11, 12]. Examination of blood samples from patients with CM have identified the upregulation of soluble levels of adhesion molecules, including intercellular adhesion molecule-1 (ICAM-1), and the dysregulation of endothelial surface markers as risk factors for complications associated with CM [13, 14]. Controlled experimental conditions are necessary to translate these findings into opportunities for testing and validating the safety and efficacy of adjunctive therapeutics that can prevent or reduce BBB dysfunction and brain injury in CM. In vitro models representing the BBB in *P. falciparum* infections can serve as tools for such investigations.

In vitro models of the BBB to study the pathogenesis of CM have been developed using porcine or rodent BMECs co-cultured with peripheral blood mononuclear cells stimulated by *Pf-*iRBCs have demonstrated BBB damage and change in expression of endothelial activation markers [15, 16]. However, porcine and rodent BMECs do not constitute direct orthologs of human BMECs. Human-derived immortalized BMECs have shown promise in in vitro models with evidence of *Pf*-iRBCs binding to cellular adhesion molecules (ICAM-1 and VCAM-1), and endothelial cell receptors (EPCR) resulting in increased permeability of human-derived immortalized BMECs [17–19]. A limitation of using immortalized cells in in vitro BBB models is reduced barrier integrity as determined by lower trans-endothelial resistance (TEER) values, ranging from 10–100 Ω x cm^2^ [20], compared to resistance values observed in human-derived primary cell based in vitro BBB models (~ 400 Ω x cm^2^) [21], likely driven by reduced expression of TJ proteins on immortalized cells [22–24]. Primary human BMECs are the gold standard in in vitro BBB models and demonstrate significantly higher barrier integrity compared to immortalized cells. Primary human BMECs have been used to study the binding of *Pf-*iRBCs to ICAM-1 and EPCR receptors on the brain’s endothelium and subsequent damage to the BBB [25–28]; and products of *P. falciparum* infections including parasite-derived histones and coagulation factor thrombin have been shown to increase BBB permeability and are toxic to BMECs [29, 30]. Nevertheless, primary BMECs are subject to batch-to-batch variability [31, 32], and their barrier properties show a decline once removed from the brain’s microenvironment [33]. Availability of primary BMECs is another limitation, and these cells are often sourced from patients with underlying pathological conditions such as epilepsy, which may alter the barrier phenotype expressed by these cells [34].

Human induced pluripotent stem cells (hiPSCs) may serve as a physiologically relevant alternative to existing model systems and have proven useful in modeling brain disorders, including cerebral fungal infections and Alzheimer's Disease [35, 36]. hiPSC-derived BMECs exhibit several phenotypic characteristics distinctive of human BMECs, including increased barrier integrity determined by TEER (~ 1500 Ω x cm^2^), low passive permeability, increased expression of TJ proteins, and expression of functionally active transporters P-glycoprotein and glucose transporter protein type-1 (GLUT-1) [37–42]. These characteristics make hiPSC-derived BMECs a viable alternative for mechanistic studies of disease-mediated barrier disruption using in vitro BBB models. To date, however, no studies have shown the utility of hiPSC-derived BMECs in studying BBB disruption and brain injury in CM.

To address this gap in knowledge, we have developed a novel, reproducible in vitro model of the BBB in CM with enhanced barrier properties using hiPSC-derived BMECs. We utilized TEER, sodium fluorescein permeability assay, and immunofluorescence imaging to observe changes in barrier integrity, and western blot to determine changes in the expression of TJ proteins. Further, we investigated if damage to endothelial cells increased the expression of endothelial cell surface markers or proteins related to the angiogenic and cell stress pathways. We hypothesized that compared to uninfected RBCs, *Pf*-iRBCs co-cultured with hiPSC-derived BMECs will demonstrate compromised BBB integrity, alterations to TJ protein localization and expression, and altered expression of factors associated with brain endothelial injury.

## Methods

### *P. falciparum* cultures

*P. falciparum clone* HB3var03, donated by J. Smith (Seattle Biomedical Research Institute, Seattle, WA), was cultured in O^+^ human RBCs (Valley Biomedical, USA) at 2% hematocrit in RPMI 1640 medium with 25 mM HEPES (Cytiva, Germany) supplemented with 10% human type A-positive serum (Interstate Blood Bank), 0.5% hypoxanthine (Sigma, USA), 7.5% sodium bicarbonate (Fisher Scientific, USA), 50 mg/ml gentamicin (Gibco, USA). *Pf*-iRBCs were maintained in a gas mixture containing 90% N_2_, 5% CO_2_, and 5% O_2._ (Praxair, USA). Parasite cultures were synchronized using 5% sorbitol, and late-stage parasites were enriched using LD MACs magnetic columns (Miltenyi Biotec, Germany) according to the manufacturer’s protocol.

### Human induced pluripotent stem cell (hiPSC) differentiation into brain microvascular endothelial-like cells (BMECs)

BMECs were differentiated from human induced pluripotent stem cells (hiPSCs) as previously described [37, 38]. Briefly, IMR90 hiPSCs (WiCell, USA) were cultured between passages 40–55 and verified for pluripotency markers, array-based Pluri-Test, and G-band karyotype analysis. BMECs were differentiated following the seeding of hiPSCs and were singularized with Accutase (Life Technologies, USA), as previously described [38, 39]. hiPSCs were plated at a density of 1 × 10^5^ cells/cm^2^ on Matrigel (Corning Life Sciences, USA) coated 6-well plates (Thermo Fisher Scientific, USA). hiPSCs were maintained for 3 days at 37 °C in mTESR nutrient medium (STEMCELL Technologies, Canada). Once an optimal density of 3 × 10^5^ cells/cm^2^ was reached, the culture medium was replaced with unconditioned DMEM-F12 media (Thermo Fisher Scientific, USA) containing 0.1 mM beta-mercaptoethanol (Sigma-Aldrich, USA) and 1 × minimum essential medium non-essential amino acids (Life Technologies, USA), to initiate cell differentiation. These conditions were maintained for 6 days at 37 °C with daily media changes. Unconditioned medium was then replaced with endothelial culture medium containing human Endothelial Serum-Free Medium (hESFM, Life Technologies, USA) supplemented with 20 ng/ml basic fibroblast growth factor (bFGF, STEMCELL Technologies, Canada), 1% platelet-poor plasma-derived bovine serum (PDS, Thermo Fisher Scientific, USA), and 10 mM retinoic acid (Sigma Aldrich, USA) for 48 h. BMECs were then seeded for co-culture experiments, maintained for an additional 24 h in EC medium (+ PDS/ + bFGF), and finally switched to maintenance in EC medium (+ PDF/−bFGF) at 37 °C for the duration of the experiment. BMECs constituted > 99% of the cell types with peak barrier properties maintained for up to 4 days post subculture [43].

### Immortalized human brain capillary endothelial cell line (hCMEC/D3) culture

Immortalized hCMEC/D3 cells (Sigma Aldrich, USA) were cultured on rat-tail collagen type 1 (Sigma Aldrich, USA) coated tissue culture flasks at 37 °C in a 5% CO_2_ incubator in EndoGRO™-MV complete media kit (Sigma Aldrich, USA) supplemented with 1 ng/ml FGF-2 (Sigma Aldrich, USA). Passages 8–13 of the hCMEC/D3 cells were used during the co-culture experiments.

### Initiation of hiPSC-derived BMEC co-culture experiments

Co-culture experiments were initiated by seeding hiPSC-derived BMECs in Matrigel-coated wells in EC medium +/+ (+ PDS/ + bFGF) for 24 h, followed by maintenance in EC medium  +/- (+ PDS/−bFGF) during the experiments. Three co-culture experimental conditions were set up: (1) hiPSC-derived BMECs only (control), (2) hiPSC-derived BMECs co-cultured with uninfected RBCs, and (3) hiPSC-derived BMECs co-cultured with late-stage enriched *Pf-*iRBC. Co-cultures were maintained for 9 h with experimental monitoring at 2, 4, 6, and 9-h.

### Measurement of transendothelial electrical resistance (TEER)

The hiPSC-derived BMECs were seeded on Matrigel-coated Transwell® inserts (0.4-micron pore size) in a 24-well plate (Corning, USA) at a cell density of 1 × 10^5^ cells/insert. TEER was measured with an epithelial Volt/Ohm meter (EVOM, World Precision Instruments, Sarasota, FL, USA). TEER measurements are reported as Ω x cm^2^ calculated by subtracting the value of an unseeded insert, multiplied by 0.33 cm^2^, to account for the surface area of the insert. Measurements were recorded at 2 h before BMEC co-culture experiments, during co-culture setup (0-h) and at 2, 4, 6, and 9-h post-co-culture. TEER measurements were normalized to the baseline resistance of control conditions with BMEC only and measured from quadruplicate filters for each biological condition.

### Sodium fluorescein permeability

To determine the permeability of the hiPSC-derived BMEC barrier, sodium (Na)-fluorescein (10 mm, 376 Dalton; Sigma Aldrich, USA) was used. Following a 6-h co-culture of hiPSC-derived BMECs with RBCs and *Pf-*iRBCs, fresh EC medium  +/- was added to the Transwell® system (0.5 ml in the top chamber, 1.5 ml in the bottom chamber) with EC medium  +/- containing Na-fluorescein added to the top chamber and EC  +/- without Na-fluorescein added to the bottom chamber. The inserts were then placed back on the rotation platform at 37 °C for 1 h. 150 ml aliquots were sampled from the bottom chamber at 15, 30, and 45 min and immediately replaced with pre-warmed EC  +/- medium. At 60 min, 150 ml aliquots were sampled from both the top and bottom chambers and fluorescence was recorded using a Synergy HTX Multi-Mode reader (BioTek, USA). Permeability co-efficient (*P*_*e*_) was calculated based on the cleared volume of Na-fluorescein from the top chamber to the bottom chamber.

### Immunocytochemistry

For immunocytochemistry experiments, co-culture conditions were set up in 96-well plates at a cell density of 1 × 10^5^ cells on Matrigel-coated wells. At 4, 6, and 9-h post-co-culture, the cells were washed three times with phosphate buffer saline (PBS, Sigma Aldrich, USA) and fixed in 100% ice-cold methanol (Sigma Aldrich, USA) for 15 min at room temperature, followed by blocking with 10% goat serum for 1 h. Cells were incubated overnight with primary antibodies at 4 °C. The following primary antibodies were used: mouse anti-occludin 3F10, 1:200; mouse anti-ZO-1 1A12, 1:100; anti-mouse claudin-5, 4C3C2, 1:50; mouse anti-GLUT-1, SPM498, 1:500 (ThermoFisher, USA); mouse anti-ICAM1 BBA3, 15 ug/ml (R&D Systems, USA); mouse anti-EPCR, ab56689, 10 mg/ml (Abcam, USA); mouse anti-VCAM-1, 1.4C3, 1:100 (ThermoFisher, USA) and rabbit anti-*Plasmodium* aldolase, 1:200 (Abcam, USA). Incubation with secondary antibodies was performed for 1 h in the dark at room temperature using goat anti-mouse IgG (Alexa Fluor 488), and goat anti-rabbit IgG (Alexa Fluor 594) (ThermoFisher, USA), 1:200. Next, we added Hoechst (ThermoFisher, USA), 1:2000 dilution, and incubated for 15 min in the dark. Finally, the BMECs were washed 3 × with PBS, and 100–200 ml of PBS was added to the cells to avoid drying. Imaging was performed using Cytation5 (BioTek, USA). The microscope settings were optimized to focus on controls at 0 h and the same settings were used for all timepoints. Following staining for occludin and ZO-1, discontinuous junctions were quantified using ImageJ with a minimum of 4 fields containing approximately 150 cells/field from 2 separate differentiations used for calculating the area fraction index for occludin and ZO-1 immunofluorescence.

### Western blotting

BMECs were washed 3 × with cold PBS and lysed using ice-cold Pierce™ RIPA buffer (Thermo Fisher, USA) and Halt™ Protease and Phosphatase Inhibitor Cocktail (Thermo Fisher, USA). Lysates were quantified for protein concentrates using a BCA Protein assay kit (Pierce, USA). Gel electrophoresis was conducted using a Protean Tetra Vertical Electrophoresis Cell (Bio-Rad, USA) and transferred to Immuno-Blot PVDF membranes (Bio-Rad, USA). The membranes were washed with Tris-buffered saline containing 0.1% Tween 20 (TBST) and blocked for 1 h at room temperature using 5% non-fat dry milk dissolved in TBST. Membranes were probed with primary antibodies against occludin (1:500), ZO-1 (1:200), and actin (1:2000) overnight in a blocking solution at 4 °C. Following a washing step using 3X TBST for 10 min, the membranes were probed with horseradish peroxidase-conjugated secondary antibodies in a blocking solution for 1 h at room temperature. After the final washing step with TBST, the membranes were subjected to Chemiluminescent Western ECL detection (Thermo Scientific). Protein levels were quantified using Image J software (NIH, USA).

### Multiplex proteome profiling of angiogenic factors and cell stress markers

Cell culture supernatants and lysates were centrifuged at 500 *g* for 5 min, and aliquots were stored at − 80 C until further use. Samples from two biologically distinct experiments were pooled for analysis, and each experiment was conducted using 3 technical replicates of samples. Angiogenic factors were measured in the cell supernatant using the Proteome Profiler™ Human Angiogenesis Antibody Array, and cell stress markers were measured in cell lysates using the Proteome Profiler™ Human Cell Stress Array (R&D system, USA) according to the manufacturer’s protocols. Briefly, 500 ml of supernatant or 150 mg of cell lysate was incubated with a cocktail of biotinylated detection antibodies and added to the array membrane. Following incubation on a rocker at 4 °C overnight, capture antibodies spotted in duplicate on the array were visualized using chemiluminescent detection reagents. The signal produced by each spot is proportional to the amount of bound analyte. The mean pixel intensity of the duplicate spots on the membrane was calculated and averaged using ImageJ Software.

### Statistical tests

All the experiments were conducted using hiPSC-derived BMECs from three differentiations, each repeated in at least technical triplicates. Two-tailed Student’s *t* test was used for comparisons between two groups. Statistical analyses were performed using one-way analysis of variance (ANOVA) from pooled data using GraphPad Prism 10.0 software. The results are presented as mean ± SEM. Mean difference in TEER values were determined using two-way ANOVA analysis. The a priori P value < 0.05 was considered statistically significant.

## Results

### Reduced barrier resistance of hiPSC-derived BMECs at 6-h post co-culture with *Pf*-iRBCs

The enhanced barrier properties of hiPSC-derived BMECs compared to immortalized hCMEC/D3 cells have been characterized in previous studies [23, 38, 41, 44]. We have included a validation of these findings in Additional file 1: Fig. S1. Briefly, TEER measurements for hiPSC-derived BMECs showed a 12-fold increase in resistance (1200 Ω x cm^2^) compared to hCMEC/D3 cells (100 Ω x cm^2^) (Additional file 1: Fig. S1A). Immunocytochemical staining of immortalized hCMEC/D3 cells showed low-level expression of TJ proteins, ZO-1 and occludin, not localized to the periphery (Additional file 1: Fig. S1B). In contrast, hiPSC-derived BMECs showed a strong signal and sharp staining pattern for ZO-1 and occludin, indicating the localization along the peripheral cell surfaces (Additional file 1: Fig. S1C), and strong expression of glucose transporter protein GLUT-1 (Additional file 1: Fig. S1D).

**Fig. 1:**
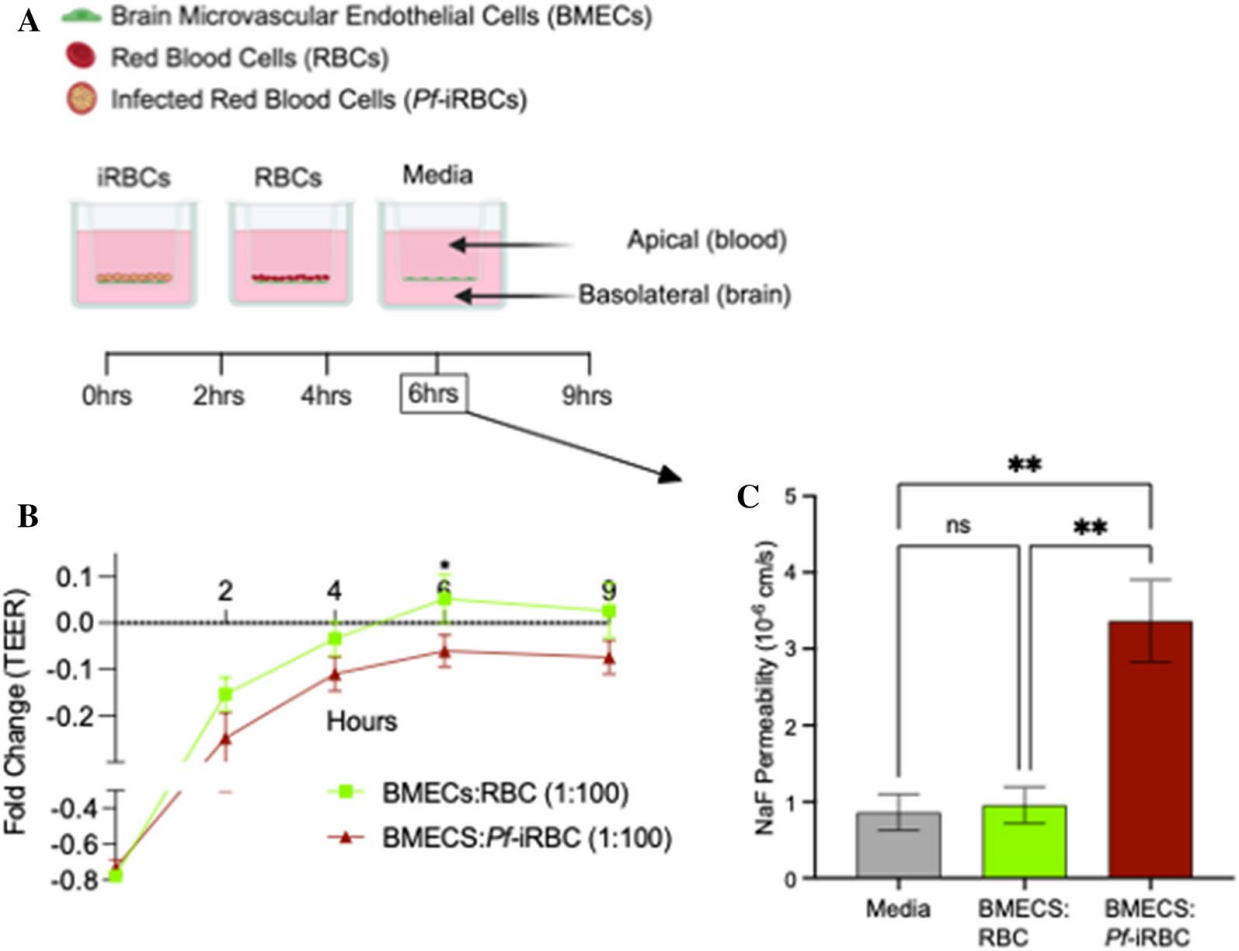
Measurement of barrier tightness (TEER) and permeability (Na-fluorescein). **A** Schematic of the experimental design (created with Biorender). **B** hiPSC-derived BMECs co-cultured with RBC (green) and *Pf-*iRBCs (red) over 2, 4, 6, and 9 h. Y-axis represents fold change in TEER compared to baseline levels in hiPSC-derived BMECs only. Ratio 1:100. **C** Sodium (Na)-fluorescein permeability measured 6 h post-co-culture. Permeability coefficients were calculated based on cleared volume of Na-fluorescein from top chamber vs. bottom chamber. Statistical significance (P < 0.05) determined by ANOVA. *P < 0.05, **P < *0.005*

The experimental design of our hiPSC-derived BMEC co-culture studies is shown in Fig. 1A. Immediately after hiPSC-derived BMECs are placed in co-culture with RBCs, there is a drop in electrical resistance, which returns to the normalized resistance level of hiPSC-derived BMECs by 4 h post co-culture (Fig. 1B). In hiPSC-derived BMECs co-cultured with *Pf-*iRBCs at a 1:100 ratio, the same recovery is observed up to 4 h (Fig. 1B). However, at 6 h post co-culture, after normalizing TEER values to those of BMECs only, there is a 10–15% decrease in electrical resistance of hiPSC-derived BMECs co-cultured with *Pf-*iRBCs compared to uninfected RBC co-cultures (P = 0.04) (Fig. 1B). At 9 h, TEER values remain lower in BMEC co-cultured with *Pf-*iRBCs compared to uninfected RBCs, although the difference was not statistically significant (P = 0.07) (Fig. 1B). A ratio of 1:50 *Pf-*iRBCs is frequently used in existing models of the BBB in CM [26]. Using a 1:50 ratio in our co-culture experiments, we observed a trend towards lower resistance in *Pf-*iRBC co-cultures at 6- and 9-h post co-culture, however, the difference compared to uninfected RBC co-cultures were not significant at any time point (all P ≥ 0.25) (Additional file 2: Fig. S2). In further validation of our model, we observed an increase in paracellular Na-fluorescein permeability in *Pf-*iRBCs co-cultures at the 6-h timepoint as determined by a higher permeability quotient (*P*_*e*_*,* 3.36 ± 0.76 × 10^–6^ cm/s) compared uninfected RBCs (0.95 ± 0.36 × 10^–6^ cm/s) or BMECs alone in culture media (0.85 ± 0.33 × 10^–6^ cm/s; all P ≤ 0.05) (Fig. 1C).

### TJ protein delocalization of on hiPSC-derived BMECs at 6 h post co-culture with *Pf*-iRBCs

**Fig. 2:**
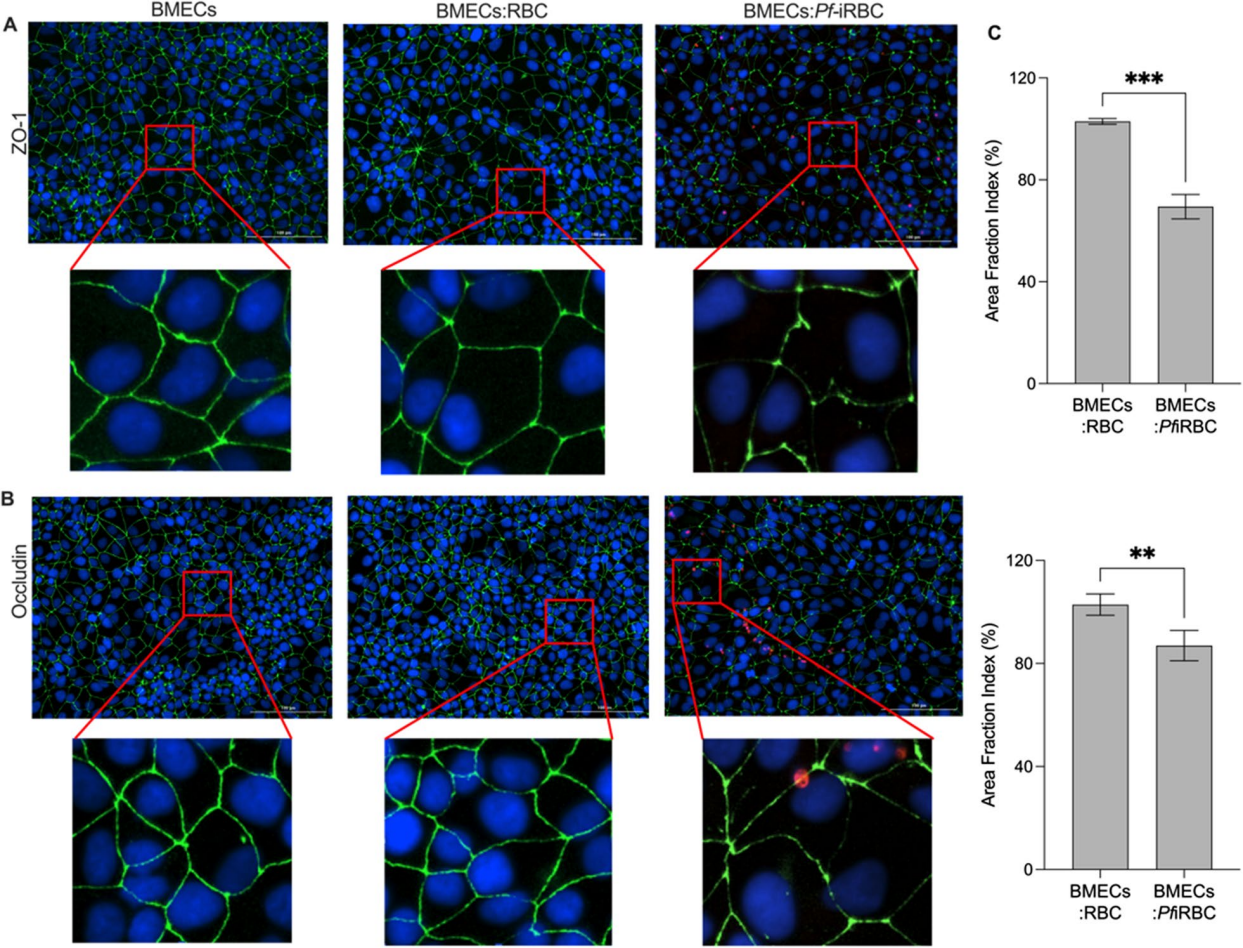
Localization of TJ proteins at 6-h post co-culture. **A** ZO-1 localization on hiPSC-derived BMECs only, BMECs co-cultured with RBCs and BMECs co-cultured with *Pf-*iRBCs. **B** Occludin localization on BMECs only, BMECs co-cultured with RBCs and BMECs co-cultured with *Pf-*iRBCs. Scale bar = 100 µm. Red boxes indicate breaks observed in TJ localization (digital expansion zoomed in on breaks in localization). ZO-1 & occludin (green), nucleus (blue), and *P.falciparum-*aldolase (red). **C** Quantification of discontinuous tight junctions for ZO-1 and occludin, using area fraction index (%). Values are normalized to BMECs only and presented as mean (SEM) of three replicates from a single differentiation and experiments repeated in two independent rounds of BMEC differentiation. *P < 0.05, **P < 0.005, ***P < *0.0005*

We investigated changes in the localization of ZO-1, occludin, and claudin-5 in hiPSC-derived BMECs in co-culture with *Pf*-iRBCs compared to uninfected RBCs at the 6-h timepoint, and quantified TJ localization by calculating the immunoreactive TJ protein signal, giving us the percent area fraction index (Fig. 2, Additional file 3: Fig. S3). There were quantifiable disruptions in the localization of ZO-1 (Fig. 2A, C), occludin (Fig. 2B, C), and claudin-5 (Additional file 3: Fig. S3A, B) in hiPSC-derived BMECs co-cultured with *Pf-*iRBCs compared to uninfected RBCs as well BMECs alone. Evidence of *Pf-*iRBCs in co-culture are shown with *Plasmodium-*specific aldolase protein staining (Fig. 2A, B), with additional evidence of RBCs and *Pf-*iRBCs shown using brightfield images taken at 6-h post co-culture (Additional file 4: Fig. S4).

In further confirmation of the trend observed with TEER values at 4- and 9-h post co-culture, the TJ breaks were not present at 4 h (Additional file 5: Fig. S5A, C); but at 9 h, the breaks in TJ proteins remained comparable to the 6-h timepoint (Additional file 5: Fig. S5B, D). There was no difference in TJ protein levels between hiPSC-derived BMECs in co-culture with uninfected RBCs compared to BMECs alone at any timepoint. We used western blotting to determine if the disruption of localization of TJ proteins was driven by changes in the expression of TJ proteins and quantified the amount of protein in the cell lysates. There was a non-significant reduction in the expression of ZO-1 and occludin at 6 h in hiPSC-derived BMECs co-cultured with *Pf*-iRBCs compared with uninfected RBCs (Additional file 6: Fig. S6).

### Increased expression of endothelial cell surface markers on hiPSC-derived BMECs co-cultured with *Pf*-iRBCs

**Fig. 3:**
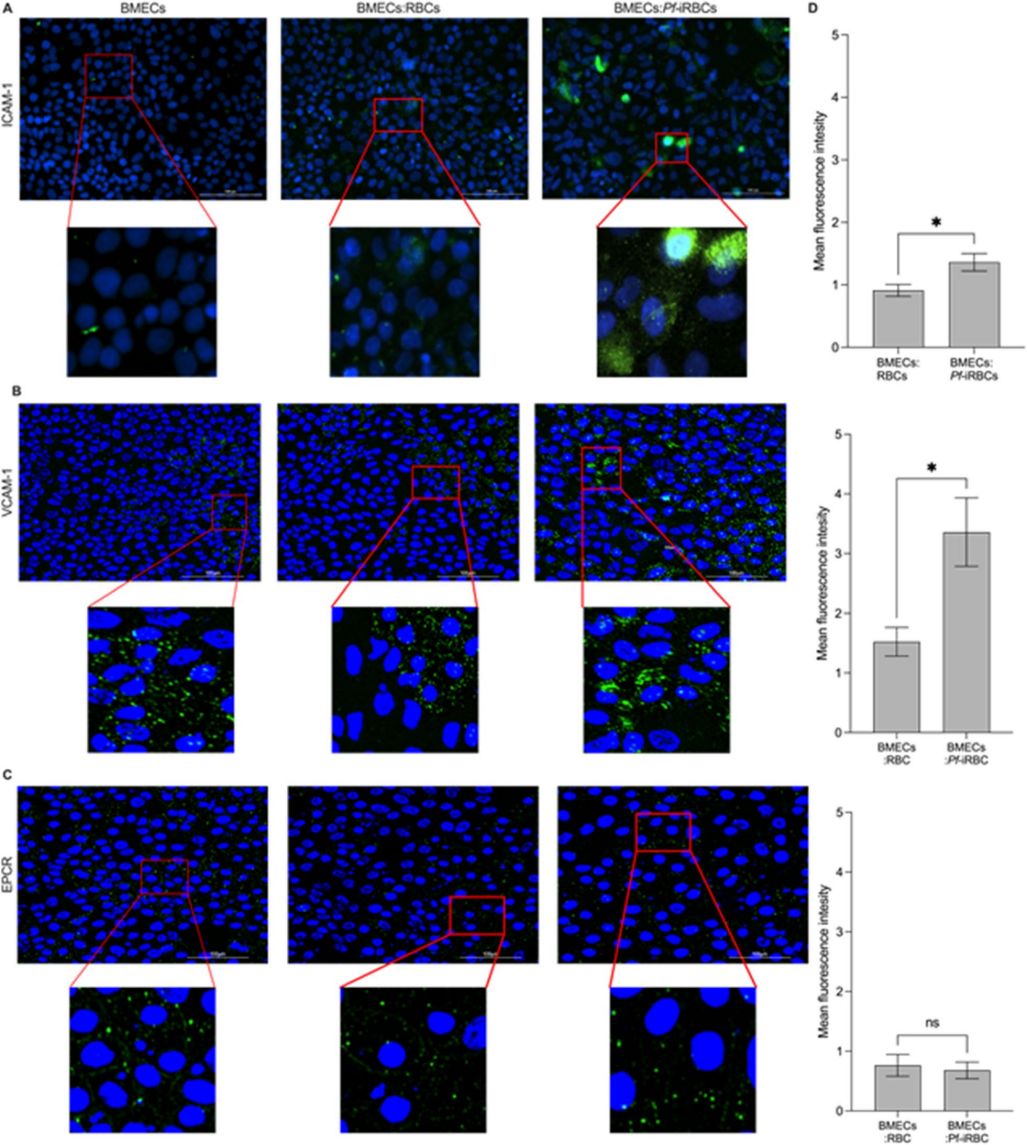
ICAM-1, VCAM-1, and EPCR expression at 6-h post co-culture. **A** ICAM-1 expression on hiPSC-derived BMECs only, BMECs co-cultured with RBCs and BMECs co-cultured with *Pf*-iRBCs. **B** VCAM-1 expression on hiPSC-derived BMECs only, BMECs co-cultured with RBCs and BMECs co-cultured with *Pf*-iRBCs. **C** EPCR expression on hiPSC-derived BMECs only, BMECs co-cultured with RBCs and BMECs co-cultured with *Pf*-iRBCs. **D** Semi-quantification to show differences in ICAM-1, VCAM-1, and EPCR expression when BMECs are co-cultured with *Pf-*iRBCs compared to RBCs normalized to baseline levels in BMECs alone. Quantification done with two independent hiPSC-derived BMEC differentiations. Immunofluorescence labeled as ICAM-1, VCAM-1, and EPCR (green), nucleus (blue) Scale bar = 100 µm. The red boxes are digitally zoomed images. Black bars represent mean ± SEM. *P < *0.05*

In addition to TJ protein localization, we investigated the change in expression of endothelial cell surface markers ICAM-1, VCAM-1, and EPCR and found increased ICAM-1 (Fig. 3A, D) and VCAM-1 (Fig. 3 B, D) expression in hiPSC-derived BMECs co-cultured with *Pf-*iRBC at 6 h compared to uninfected RBC co-cultures or BMECs alone. As shown in prior studies [18], ICAM-1 expression was observed within and on the surface of the hiPSC-derived BMECs (Fig. 3A). There was no difference in the expression of EPCR in either condition (Fig. 3C, D).

### Changes in the expression of markers of angiogenesis and cellular stress in hiPSC-derived BMECs co-cultured with *Pf*-iRBCs

**Fig. 4:**
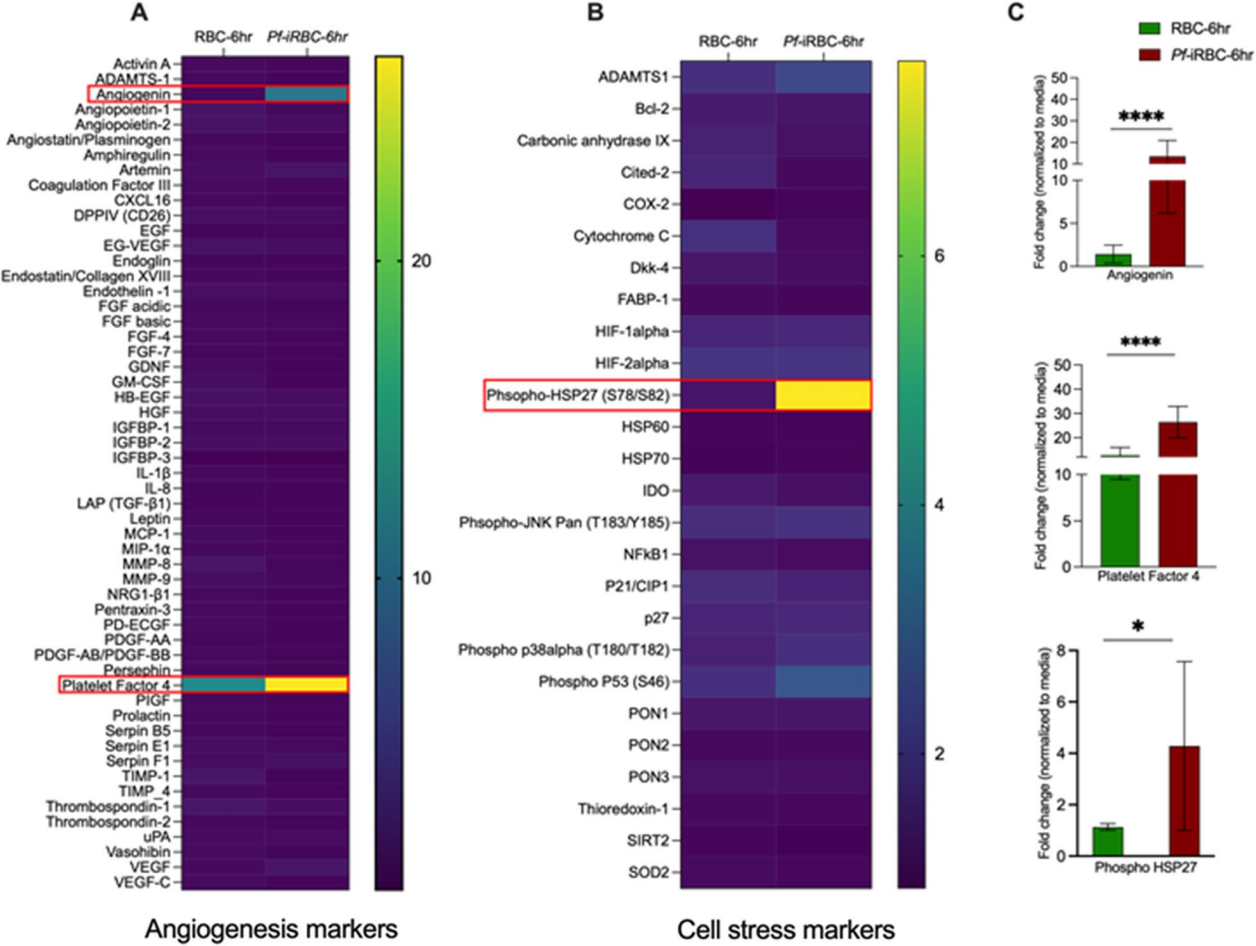
Expression of angiogenic and cell stress markers at 6-h post co-culture. **A** Heat map of fold change in expression of angiogenic markers (mean pixel intensity) in hiPSC-derived BMECs co-cultured with RBCs and *Pf-*iRBCs **B** Heat map of fold change in expression of cellular stress markers (mean pixel intensity) in hiPSC-derived BMECs co-cultured with RBCs and *Pf-*iRBCs. Red boxes highlight proteins with significant difference in expression between hiPSC-derived BMECs co-cultured with RBCs vs *Pf-*iRBCs. **C** Fold-change in protein expression in RBCs co-culture (green) compared to *Pf*-iRBC co-culture (red) normalized to baseline protein expression in BMECs alone, calculated from data obtained from experiments from two independent hiPSC-derived BMEC differentiation. *P < 0.05, ****P < *0.00005*

We investigated whether *Pf*-iRBCs can alter the secretion patterns of angiogenic and cell stress markers on hiPSC-derived BMECs. Using a panel of 55 angiogenesis markers, we observed a tenfold increase in the expression of angiogenin and a 25-fold increase in the expression of platelet factor 4 (PF4) at 6 h in the supernatant from hiPSC-derived BMECs co-cultured with *Pf-*iRBCs compared with uninfected RBCs (Fig. 4A, C). No other angiogenic marker was significantly altered in the co-culture supernatants. Among the 26 cellular stress markers analyzed in the cell lysate, there was a fourfold increase in production of cellular stress marker, phospho-heat shock protein 27 (phospho-HSP27), in *Pf-*iRBCs co-cultures compared with RBCs (Fig. 4B, C). Immunoblot images are shown in Additional file 7: Fig. S7.

## Discussion

In this study, we demonstrate that human stem cell-derived brain microvascular endothelial cells can serve as a physiologically relevant model of the BBB in cerebral malaria. We show increased barrier tightness, as determined by TEER in our hiPSC-derived BMECs compared to an established immortalized hCMEC/D3 cell line. The expression and membrane-specific localization of BBB-specific TJ proteins, ZO-1, occludin, and claudin-5, and expression of glucose transporter protein, GLUT-1 on hiPSC-derived BMECs confirms the validity of our hiPSC-BMECs based model. In contrast, immortalized hCMEC/D3 cells showed no expression of occludin, and cytosolic localization of expressed ZO-1 and not on the periphery. When co-cultured with *Pf-*iRBC, we show evidence of key features involved in the pathogenesis of human cerebral malaria, including a decrease in TEER, increase in paracellular sodium fluorescein permeability, and disruption of ZO-1, occludin, and claudin-5 localization after 6 h of co-culture. These changes were not observed in hiPSC-derived BMECs co-cultured with uninfected RBCs. Further, ICAM-1 and VCAM-1 expression was higher in *Pf-*iRBC co-cultures compared to uninfected RBCs. Finally, increased expression of angiogenic growth factors and cellular stress markers, provide additional evidence of disruption of the BBB mediated specifically by *Pf-*iRBCs in our in vitro model. Taken together, our findings confirm the validity and usefulness of a novel, in vitro BBB model with enhanced barrier integrity that can be used to study the pathogenesis of brain injury in CM.

Immortalized BMECs are routinely used to study drug permeability across the BBB, although they have been shown to lack the requisite barrier tightness [23, 45]. Immortalized hCMEC/D3 are also used in in vitro models of the BBB in CM [18, 44, 46]. In comparison to the low TEER of immortalized hCMEC/D3 cells (100 Ω x cm^2^), hiPSC-derived BMECs used in this study demonstrated significantly higher TEER (1200 Ω x cm^2^), and proper localization and expression of TJ proteins. The barrier integrity of hiPSC-derived BMECs exposed to *Pf-*iRBCs was significantly reduced after 6-houirs of co-culture compared to uninfected RBCs, indicating parasite mediated disruption of the BBB. This was further confirmed by the increased sodium fluorescein permeability observed after 6 h of co-culture with *Pf-*iRBCs compared to uninfected RBCs. It has been proposed that unlike the findings of complete BBB breakdown shown in in vitro and in vivo experimental models of CM, barrier breakdown in human CM infections is likely partial, resulting from TJ delocalization [47]. Indeed, while we observed disruption of TJ protein localization at 6 h in hiPSC-derived BMECs in co-culture with *Pf*-iRBCs, there was no evidence of change in the expression of TJ proteins, consistent with in vivo findings that BBB breakdown in CM may be partial. Additional studies with longer co-culture timepoints are needed to corroborate the findings of this study. In our experimental setup, we were unable to maintain the integrity of co-culture conditions beyond 9 h. Notwithstanding, the barrier tightness, and the membrane surface localization of TJ proteins demonstrated by hiPSC-derived BMECs in our study make a compelling case for their use in malaria-related drug permeability studies.

The parasite HB3var03 strain used in our study was selected for its ability to bind brain endothelial cell receptors ICAM-1 and EPCR [27], designed to serve as a surrogate for *P. falciparum* infections leading to clinical CM. A recent study investigating BBB breakdown used the HB3var03 strain to show binding of *Pf-*iRBCs to brain endothelial cells in an ICAM-1-dependent manner [18]. In concordance with these findings, in our study, there was an HB3var03 strain *Pf*-iRBC driven increase in the expression of ICAM-1 and VCAM-1 on hiPSC-derived BMECs that was not observed in uninfected RBC co-cultures. There was no significant difference in EPCR expression levels in either experimental condition, which corroborates previous studies [48]. Additional studies to further validate our model using different parasite strains are needed including those that express other var genes encoding EPCR-binding membrane protein (*Pf*EMP-1) variants associated with disease severity in CM [49].

In a novel finding, levels of angiogenic factors, angiogenin and PF4, and cell stress marker, phospho-HSP27 were significantly higher in hiPSC-derived BMECs co-cultured with *Pf-*iRBCs compared to uninfected RBCs co-cultures. Reports on disorders such as ischemia and small vessel disease have shown damage to brain endothelial cells can result in the increase in expression of angiogenic and cell stress factors associated with cellular injury [50, 51]. Exogenous expression of angiogenin has been implicated in endothelial damage and accelerated angiogenesis [52–54]. PF4 is released in an inflammatory response to thrombocytopenia, primarily by activated platelets, although endothelial cells have been shown to produce PF4 as well [55]. Platelets play a dual role in malaria pathogenesis, with their homeostatic function contributing to infection control in malaria, whereas activated platelets are involved in the pathogenesis of disease severity in CM [56]. In in vitro malaria studies, PF4 has been shown to play a protective role in platelet-mediated killing of *Pf-*iRBCs [57]. In contrast, PF4 released from activated platelets appears to promote the development of CM in experimental mouse models [58]. Beyond malaria, in vitro and mouse model studies on vascular permeability, leukocyte recruitment, and endothelial cell signaling have shown PF4 to cause increased endothelial permeability in response to cellular damage [59]. These reports, and our findings of elevated angiogenin and PF4 in *Pf*-iRBCs co-cultured with BMECs, warrants further investigations on their roles in mediating cellular damage in severe falciparum malaria. Further, phospho-HSP-27 has been shown to protect damaged endothelial cells in ischemia models via cytoskeletal actin rearrangement in response to ischemic injury [60–62]. Increased expression of phospho-HSP27 in response to barrier disruption in *Pf-*iRBC co-culture may serve to repair the parasite-mediated damage to hiPSC-derived BMECs. This may explain the trend towards recovery observed at 9-h post co-culture in our study. Additional studies to validate these findings are needed. If confirmed, it would suggest a therapeutic potential for phospho-HSP27 to interrupt endothelial damage and mediate BBB recovery in CM and other brain injury disorders.

Among the limitations of our study are that co-culture conditions could only be sustained for up to 9 h and using a single cycle of synchronized *Pf*-iRBCs at a fixed ratio of BMECs to *Pf*-iRBCs, making it less representative of human CM infections, which are asynchronous with steadily increasing parasite densities when left untreated. Nevertheless, we optimized our experimental conditions to capture human infections as closely as possible, using a 1:100 ratio of BMECs to *Pf*-iRBCs, to mimic hyperparasitemia observed in severe malarial infections. Coma is a defining feature of CM, but it is not possible to reproduce the clinical features of CM in a laboratory model. Animal models may be more useful in replicating clinical complications associated with CM, but current experimental animal models of cerebral malaria rely on non-falciparum malaria species for infection that differ in their cytoadherence and infection patterns. Our in vitro model, which utilizes human-derived brain endothelial cells and *P. falciparum* parasite strains provides a suitable approximation to study certain pathways and mechanisms associated with parasite-mediated BBB disruption in a controlled environment. It can be particularly valuable for drug permeability studies for adjunctive therapies to prevent or reduce brain injury in CM. This proof-of-concept study was limited to investigating the interaction of *Pf*-iRBCs with BMECs and validating the usefulness of hiPSC-derived BMECs as a surrogate for studying the BBB in CM. However, the neurovascular unit that forms the BBB is comprised of multiple cell types, including pericytes and astrocytes, and immune cells, inflammatory factors, and parasite toxins are known contributors to BBB disruption in CM. Our in vitro model provides a foundation for future studies utilizing hiPSC-derived cells of the neurovascular unit for a more comprehensive evaluation of the pathogenic mechanisms underlying *P. falciparum* infections.

## Conclusion

Despite the availability of useful preventative and treatment strategies, malaria continues to be one of the leading causes of death and disability in Africa, disproportionately affecting the youngest children. There remains a critical lack of adjunctive therapies to prevent mortality in the sickest children. Our limited understanding of the pathogenesis of brain injury in CM, in part, due to the difficulty of directly studying injury to the brain in severely ill children, is an important driver of this knowledge gap. Our novel hiPSC-derived model of the BBB with enhanced barrier properties is a useful step in improving our understanding of how *Plasmodium falciparum-*infected RBCs sequestered to the brain's endothelium leads to the disruption of the blood–brain barrier involved in protecting the delicate structure of the neurovascular unit from systemic damage. Our model shows promise for the study of potential adjunctive neuroprotective therapies to prevent or reduce future neurocognitive defects observed in survivors of CM.

### Supplementary Information

**Additional file 1: Figure S1.** Localization of tight junction (TJ) proteins and TEER measurements in immortalized hCMEC/D3 and hiPSC-derived BMECs. (A) TEER measurements from hCMEC/D3 & hiPSC-derived BMECs. (B) ZO-1 and occludin localization on hCMEC/D3 cells. (C) ZO-1 and occludin localization on hiPSC-derived BMECs (D) GLUT-1 localization in hiPSC-derived BMECs. Immunofluorescence labeled as ZO-1, occludin, and GLUT-1 (green), nucleus (blue). Scale bar = 100 µm.

**Additional file 2: Figure S2.** Effect of *Pf-*iRBCs and RBCs on barrier tightness in hiPSC-derived BMECs over a period of 2, 4, 6, and 9 h. Fold change when hiPSC-derived BMECs were exposed to *Pf-*iRBC and RBCs in a ratio of 1:50, respectively.

**Additional file 3: Figure S3.** Localization of claudin-5 protein in hiPSC-derived BMECs co-cultured with RBCs or *Pf-*iRBCs at 6-h post co-culture. (A) Claudin-5 localization in hiPSC-derived BMECs co-cultured with RBCs and *Pf-*iRBCs. Immunofluorescent images are shown with claudin-5 in green. (B) Quantification of discontinuous tight junctions for claudin-5, using area fraction index (%). Values are normalized to hiPSC-derived BMECs only and presented as mean ± SEM of three replicates from a single differentiation and experiments were repeated in two independent rounds of iPSC-derived BMEC differentiation. *P < *0.05.*

**Additional file 4: Figure S4.** Localization of TJ proteins at 4- and 9-h post co-culture. Immunofluorescence labeled as the nucleus (blue), ZO-1 & occludin (green) and *P. falciparum* (red). (A & B) ZO-1 expression in hiPSC-derived BMECs co-cultured with RBCs and *Pf-*iRBCs. (C & D) Occludin expression in hiPSC-derived BMECs co-cultured with RBCs and *Pf-*iRBCs. The red box indicates discontinuous junctions. Digital zoomed images show these breaks. Scale bar = 100 µm.

**Additional file 5: Figure S5.** Localization of TJ proteins at 4- and 9-h post co-culture. Immunofluorescence labeled as the nucleus (blue), ZO-1 & occludin (green) and *P. falciparum* (red). (A & B) ZO-1 expression in hiPSC-derived BMECs co-cultured with RBCs and *Pf-*iRBCs. (C & D) Occludin expression in hiPSC-derived BMECs co-cultured with RBCs and *Pf-*iRBCs. The red box indicates discontinuous junctions. Digital zoomed images show these breaks. Scale bar = 100 µm

**Additional file 6: Figure S6.** TJ protein expression by western blot. ZO-1 and occludin expression shown along side β-actin loading control. Graph represents fold-change quantification on the Y-axis of ZO-1 and occludin expression against the expression on hiPSC-derived BMECs alone normalized to β-actin control. Values are presented as mean (SEM) of three independent differentiations.

**Additional file 7: Figure S7.** Angiogenesis and cell stress panels tested at 6 h post co-culture. (A) Angiogenesis markers measured in cell supernatamt. (B) Cell stress markers measured in cell lysates. Panels include hiPSC-derived BMECs only, hiPSC-derived BMECs with RBCs, and hiPSC-derived BMECs with *Pf-*iRBCs. Red and blue boxes show differences in angiogenin and platelet factor-4. White box shows differences in phospho-HSP27. Values presented as mean (SEM) from three differentiations.

## Data Availability

We confirm that all data from this study will be available upon request.

## Abbreviations

BBB: Blood–brain barrier
BMECs: Brain microvascular endothelial cells
CM: Cerebral malaria
EPCR: Endothelial protein C receptor
GLUT-1: Glucose transporter protein type-1
hiPSC: Human-induced pluripotent stem cells
HSP27: Heat shock protein 27
ICAM-1: Intercellular adhesion molecule-1
IF: Immunofluorescence
Pf: *Plasmodium falciparum*
*Pf*-iRBC: *Plasmodium falciparum* Infected red blood cells
PF4: Platelet factor 4
RBC: Red blood cells
TEER: Trans-endothelial electrical resistance
TJ: Tight junction
VCAM-1: Vascular cell adhesion molecule-1
WB: Western blot
ZO-1: Zonula occludens-1
