# Supplementary material for: A human pluripotent stem cell-derived in vitro model of the blood–brain barrier in cerebral malaria
Source: Fluids Barriers CNS. 2024 May 1;21:38. doi: 10.1186/s12987-024-00541-9 (PMC11064301; doi:10.1186/s12987-024-00541-9)
Supplement: Supplementary file 1 — Additional file 1: Figure S1. Localization of tight junction (TJ) proteins and TEER measurements in immortalized hCMEC/D3 and hiPSC-derived BMECs. (A) TEER measurements from hCMEC/D3 & hiPSC-derived BMECs. (B) ZO-1 and occludin localization on hCMEC/D3 cells. (C) ZO-1 and occludin localization on hiPSC-derived BMECs (D) GLUT-1 localization in hiPSC-derived BMECs. Immunofluorescence labeled as ZO-1, occludin, and GLUT-1 (green), nucleus (blue). Scale bar = 100 µm. [file 12987_2024_541_MOESM1_ESM.pptx]

## Slide 1
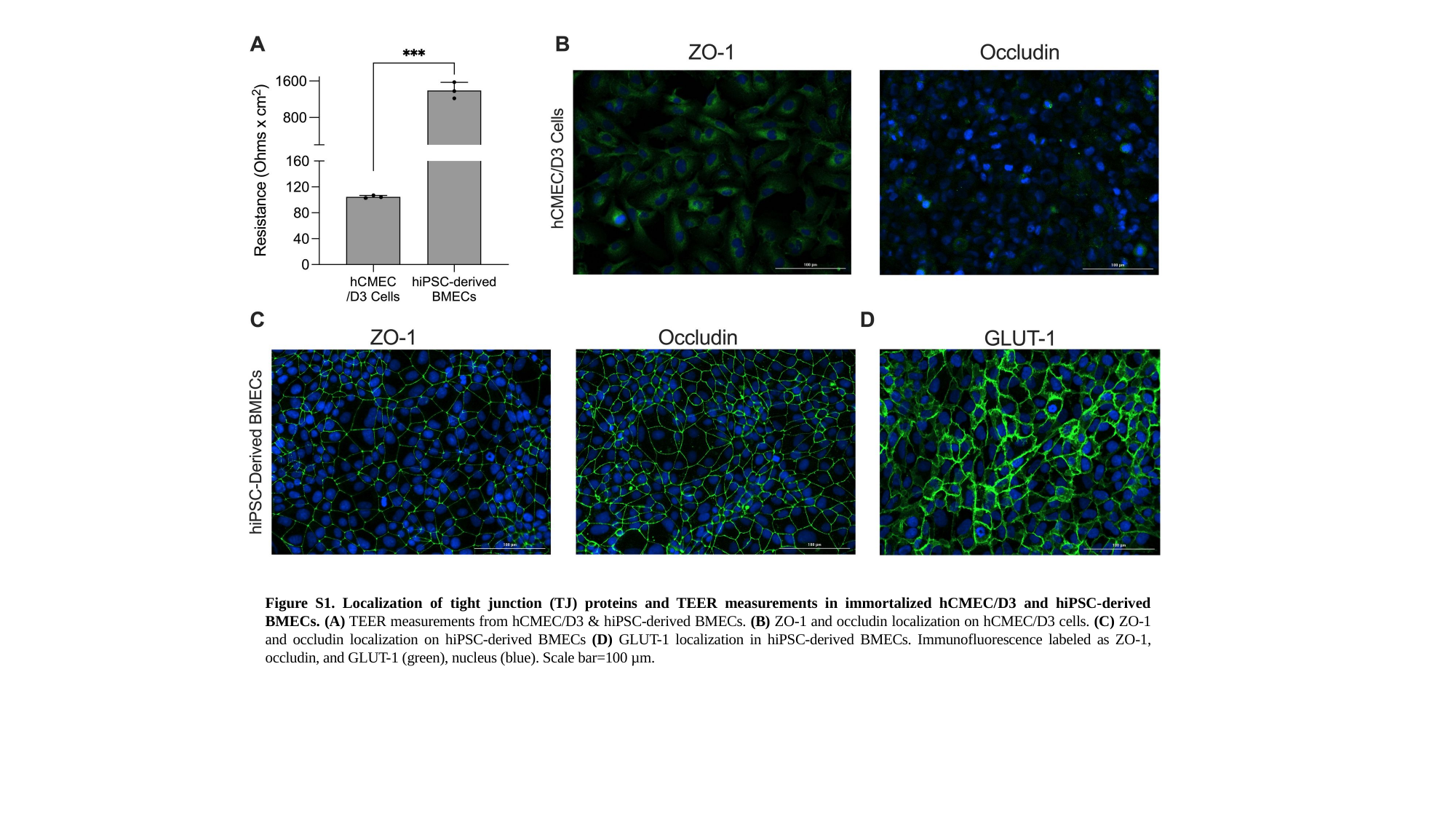

Figure S1. Localization of tight junction (TJ) proteins and TEER measurements in immortalized hCMEC/D3 and hiPSC-derived BMECs. (A) TEER measurements from hCMEC/D3 & hiPSC-derived BMECs. (B) ZO-1 and occludin localization on hCMEC/D3 cells. (C) ZO-1 and occludin localization on hiPSC-derived BMECs (D) GLUT-1 localization in hiPSC-derived BMECs. Immunofluorescence labeled as ZO-1, occludin, and GLUT-1 (green), nucleus (blue). Scale bar=100 µm.
