# Supplementary material for: A human pluripotent stem cell-derived in vitro model of the blood–brain barrier in cerebral malaria
Source: Fluids Barriers CNS. 2024 May 1;21:38. doi: 10.1186/s12987-024-00541-9 (PMC11064301; doi:10.1186/s12987-024-00541-9)
Supplement: Supplementary file 2 — Additional file 2: Figure S2. Effect of Pf-iRBCs and RBCs on barrier tightness in hiPSC-derived BMECs over a period of 2, 4, 6, and 9 h. Fold change when hiPSC-derived BMECs were exposed to Pf-iRBC and RBCs in a ratio of 1:50, respectively. [file 12987_2024_541_MOESM2_ESM.pptx]

## Slide 1
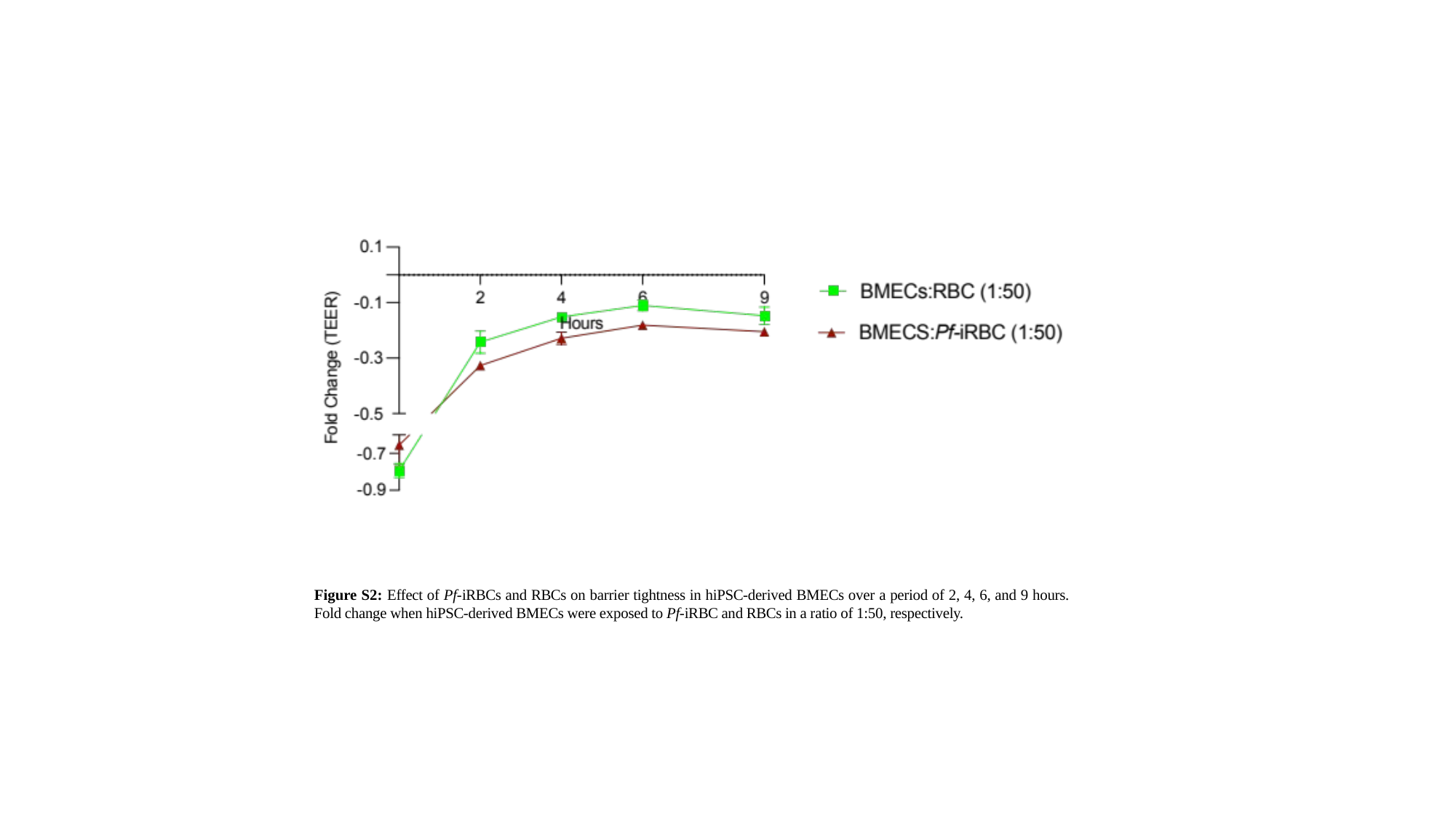

Figure S2: Effect of Pf-iRBCs and RBCs on barrier tightness in hiPSC-derived BMECs over a period of 2, 4, 6, and 9 hours. Fold change when hiPSC-derived BMECs were exposed to Pf-iRBC and RBCs in a ratio of 1:50, respectively.
