# Supplementary material for: A human pluripotent stem cell-derived in vitro model of the blood–brain barrier in cerebral malaria
Source: Fluids Barriers CNS. 2024 May 1;21:38. doi: 10.1186/s12987-024-00541-9 (PMC11064301; doi:10.1186/s12987-024-00541-9)
Supplement: Supplementary file 3 — Additional file 3: Figure S3. Localization of claudin-5 protein in hiPSC-derived BMECs co-cultured with RBCs or Pf-iRBCs at 6-h post co-culture. (A) Claudin-5 localization in hiPSC-derived BMECs co-cultured with RBCs and Pf-iRBCs. Immunofluorescent images are shown with claudin-5 in green. (B) Quantification of discontinuous tight junctions for claudin-5, using area fraction index (%). Values are normalized to hiPSC-derived BMECs only and presented as mean ± SEM of three replicates from a single differentiation and experiments were repeated in two independent rounds of iPSC-derived BMEC differentiation. *P < 0.05. [file 12987_2024_541_MOESM3_ESM.pptx]

## Slide 1
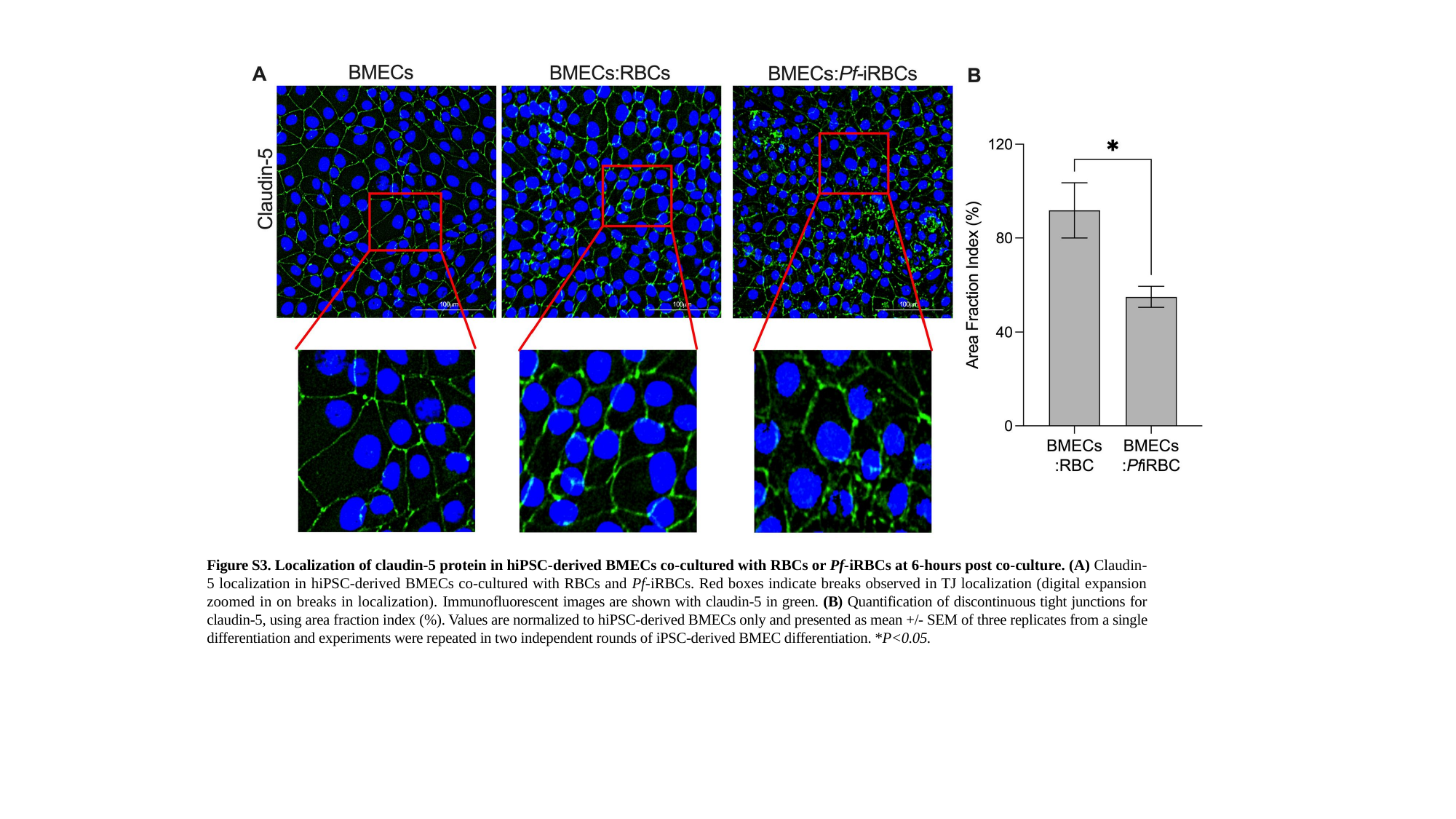

Figure S3. Localization of claudin-5 protein in hiPSC-derived BMECs co-cultured with RBCs or Pf-iRBCs at 6-hours post co-culture. (A) Claudin-5 localization in hiPSC-derived BMECs co-cultured with RBCs and Pf-iRBCs. Red boxes indicate breaks observed in TJ localization (digital expansion zoomed in on breaks in localization). Immunofluorescent images are shown with claudin-5 in green. (B) Quantification of discontinuous tight junctions for claudin-5, using area fraction index (%). Values are normalized to hiPSC-derived BMECs only and presented as mean +/- SEM of three replicates from a single differentiation and experiments were repeated in two independent rounds of iPSC-derived BMEC differentiation. *P<0.05.
