# Supplementary material for: A human pluripotent stem cell-derived in vitro model of the blood–brain barrier in cerebral malaria
Source: Fluids Barriers CNS. 2024 May 1;21:38. doi: 10.1186/s12987-024-00541-9 (PMC11064301; doi:10.1186/s12987-024-00541-9)
Supplement: Supplementary file 4 — Additional file 4: Figure S4. Localization of TJ proteins at 4- and 9-h post co-culture. Immunofluorescence labeled as the nucleus (blue), ZO-1 & occludin (green) and P. falciparum (red). (A & B) ZO-1 expression in hiPSC-derived BMECs co-cultured with RBCs and Pf-iRBCs. (C & D) Occludin expression in hiPSC-derived BMECs co-cultured with RBCs and Pf-iRBCs. The red box indicates discontinuous junctions. Digital zoomed images show these breaks. Scale bar = 100 µm. [file 12987_2024_541_MOESM4_ESM.pptx]

## Slide 1
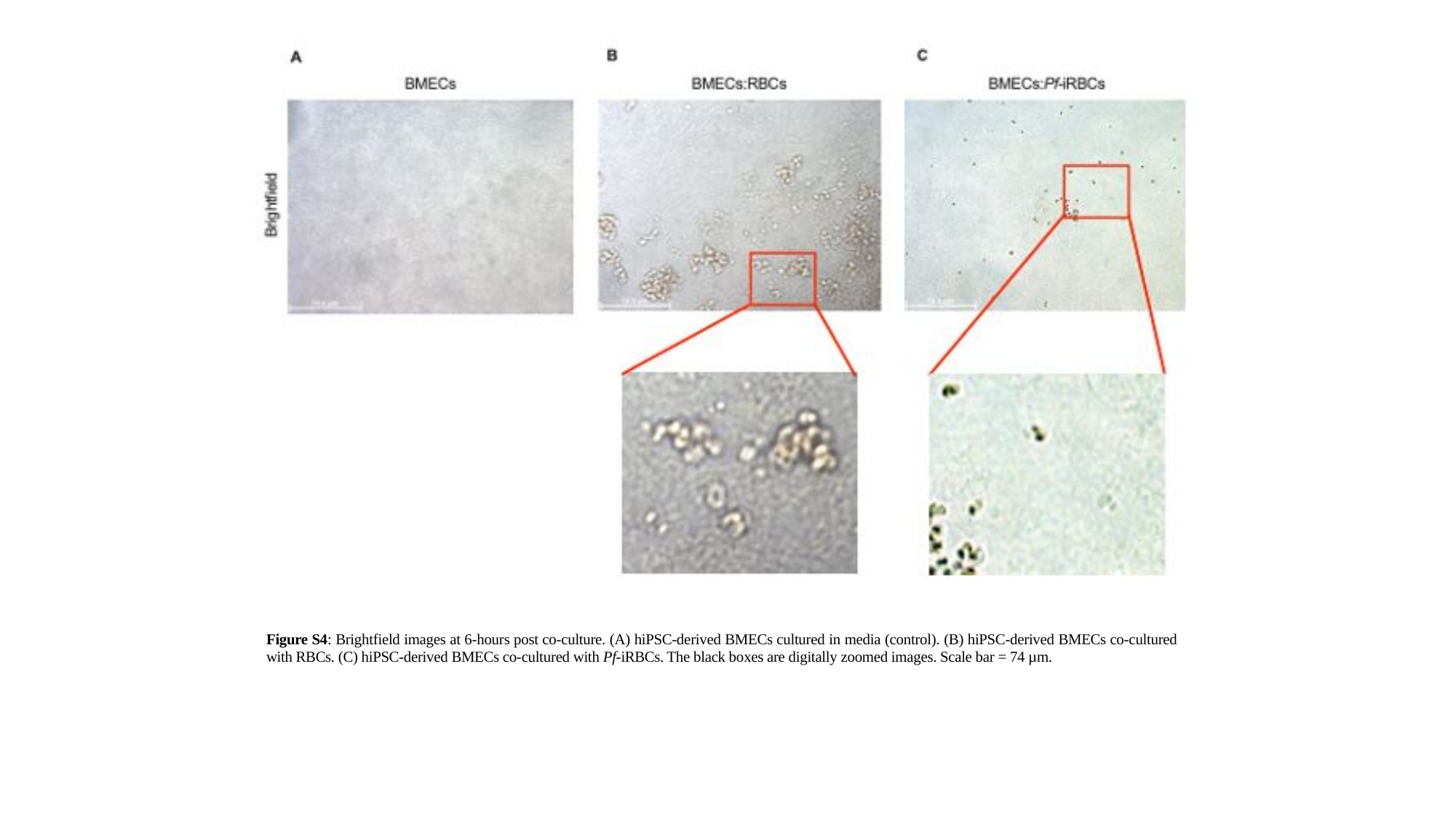

Figure S4: Brightfield images at 6-hours post co-culture. (A) hiPSC-derived BMECs cultured in media (control). (B) hiPSC-derived BMECs co-cultured with RBCs. (C) hiPSC-derived BMECs co-cultured with Pf-iRBCs. The black boxes are digitally zoomed images. Scale bar = 74 µm.
