# Supplementary material for: A human pluripotent stem cell-derived in vitro model of the blood–brain barrier in cerebral malaria
Source: Fluids Barriers CNS. 2024 May 1;21:38. doi: 10.1186/s12987-024-00541-9 (PMC11064301; doi:10.1186/s12987-024-00541-9)
Supplement: Supplementary file 5 — Additional file 5: Figure S5. Localization of TJ proteins at 4- and 9-h post co-culture. Immunofluorescence labeled as the nucleus (blue), ZO-1 & occludin (green) and P. falciparum (red). (A & B) ZO-1 expression in hiPSC-derived BMECs co-cultured with RBCs and Pf-iRBCs. (C & D) Occludin expression in hiPSC-derived BMECs co-cultured with RBCs and Pf-iRBCs. The red box indicates discontinuous junctions. Digital zoomed images show these breaks. Scale bar = 100 µm [file 12987_2024_541_MOESM5_ESM.pptx]

## Slide 1
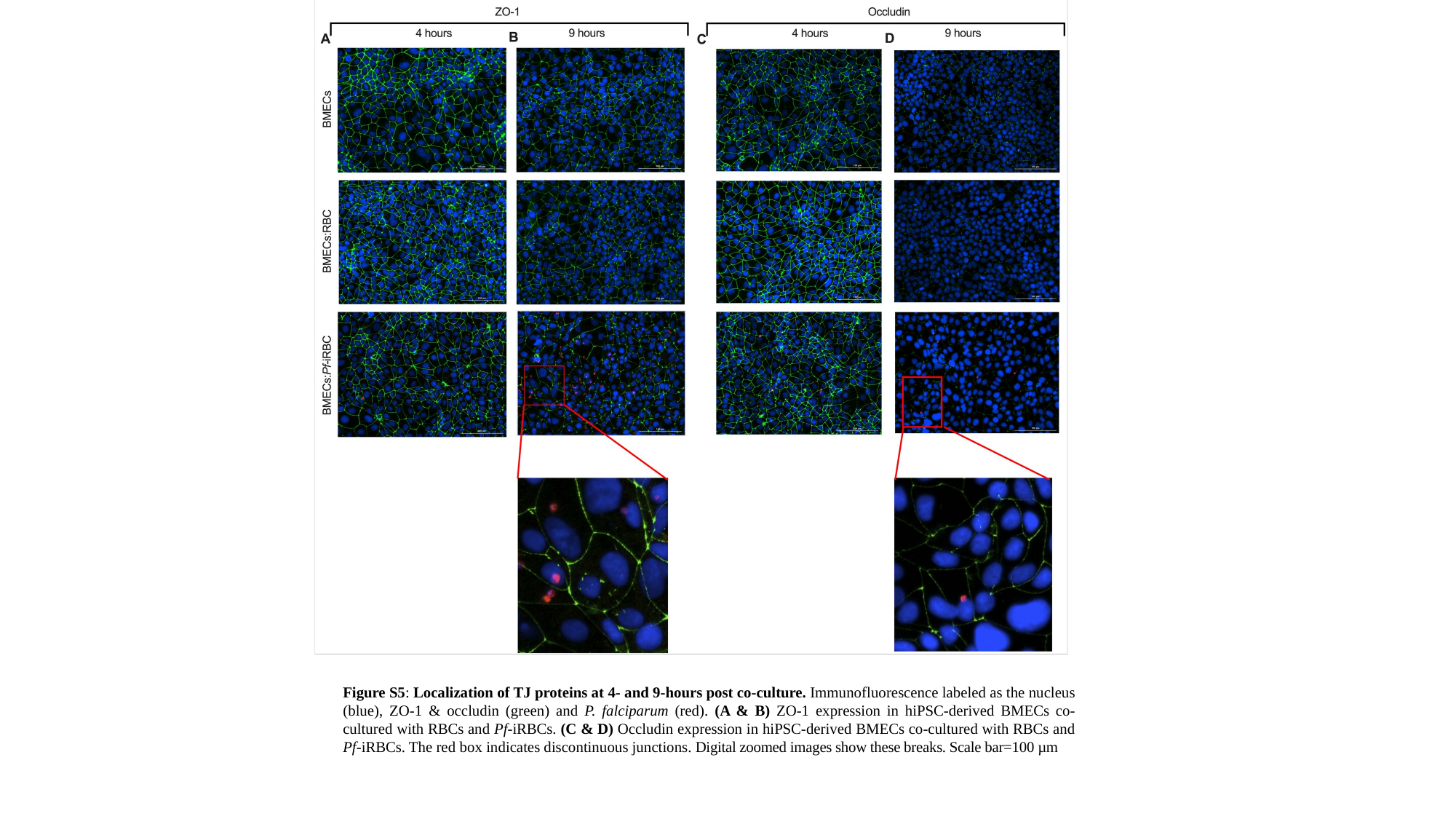

Figure S5: Localization of TJ proteins at 4- and 9-hours post co-culture. Immunofluorescence labeled as the nucleus (blue), ZO-1 & occludin (green) and P. falciparum (red). (A & B) ZO-1 expression in hiPSC-derived BMECs co-cultured with RBCs and Pf-iRBCs. (C & D) Occludin expression in hiPSC-derived BMECs co-cultured with RBCs and Pf-iRBCs. The red box indicates discontinuous junctions. Digital zoomed images show these breaks. Scale bar=100 µm
