# Supplementary material for: A human pluripotent stem cell-derived in vitro model of the blood–brain barrier in cerebral malaria
Source: Fluids Barriers CNS. 2024 May 1;21:38. doi: 10.1186/s12987-024-00541-9 (PMC11064301; doi:10.1186/s12987-024-00541-9)
Supplement: Supplementary file 6 — Additional file 6: Figure S6. TJ protein expression by western blot. ZO-1 and occludin expression shown along side β-actin loading control. Graph represents fold-change quantification on the Y-axis of ZO-1 and occludin expression against the expression on hiPSC-derived BMECs alone normalized to β-actin control. Values are presented as mean (SEM) of three independent differentiations. [file 12987_2024_541_MOESM6_ESM.pptx]

## Slide 1
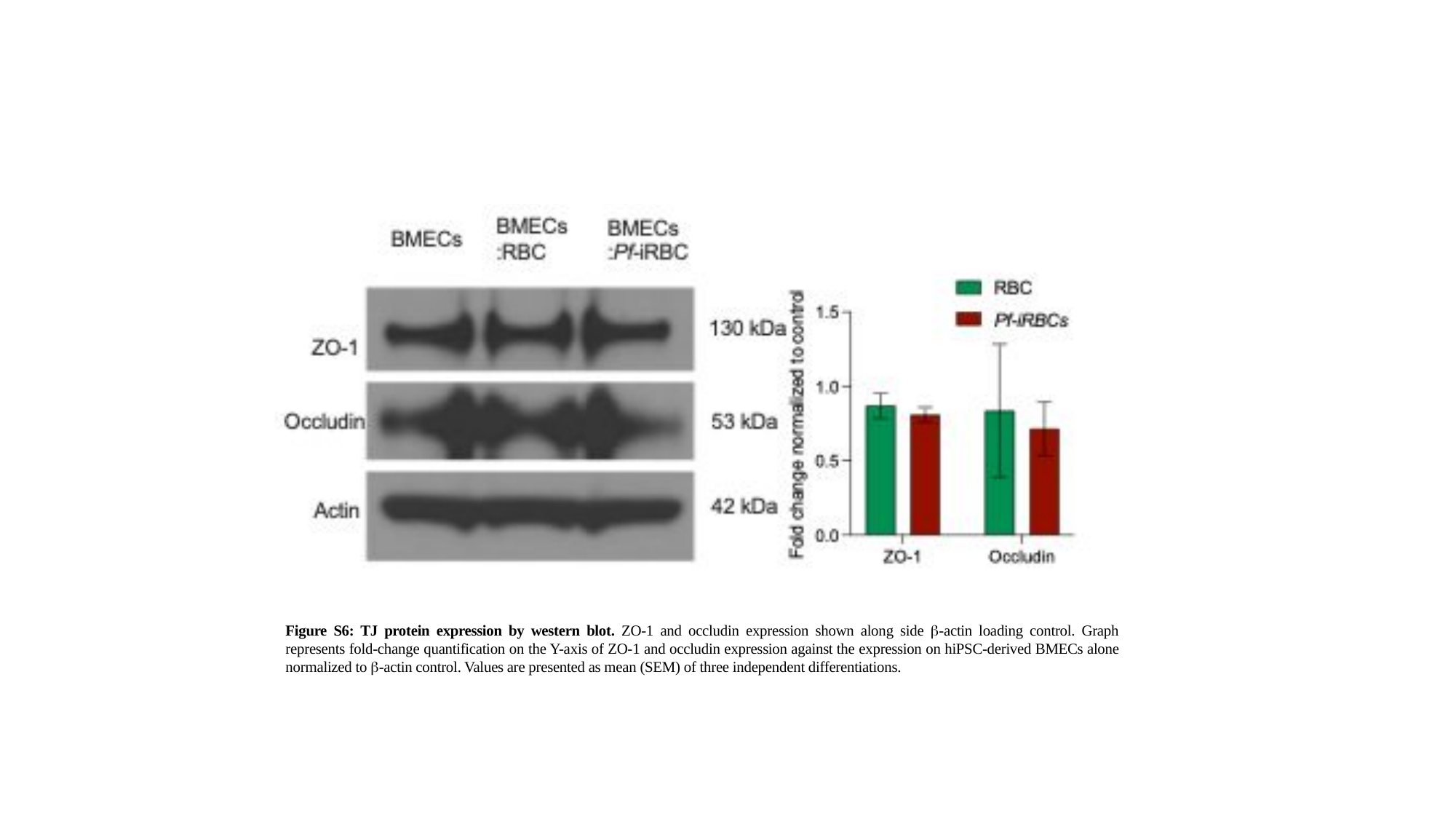

Figure S6: TJ protein expression by western blot. ZO-1 and occludin expression shown along side β-actin loading control. Graph represents fold-change quantification on the Y-axis of ZO-1 and occludin expression against the expression on hiPSC-derived BMECs alone normalized to β-actin control. Values are presented as mean (SEM) of three independent differentiations.
