# Supplementary material for: A human pluripotent stem cell-derived in vitro model of the blood–brain barrier in cerebral malaria
Source: Fluids Barriers CNS. 2024 May 1;21:38. doi: 10.1186/s12987-024-00541-9 (PMC11064301; doi:10.1186/s12987-024-00541-9)
Supplement: Supplementary file 7 — Additional file 7: Figure S7. Angiogenesis and cell stress panels tested at 6 h post co-culture. (A) Angiogenesis markers measured in cell supernatamt. (B) Cell stress markers measured in cell lysates. Panels include hiPSC-derived BMECs only, hiPSC-derived BMECs with RBCs, and hiPSC-derived BMECs with Pf-iRBCs. Red and blue boxes show differences in angiogenin and platelet factor-4. White box shows differences in phospho-HSP27. Values presented as mean (SEM) from three differentiations. [file 12987_2024_541_MOESM7_ESM.pptx]

## Slide 1
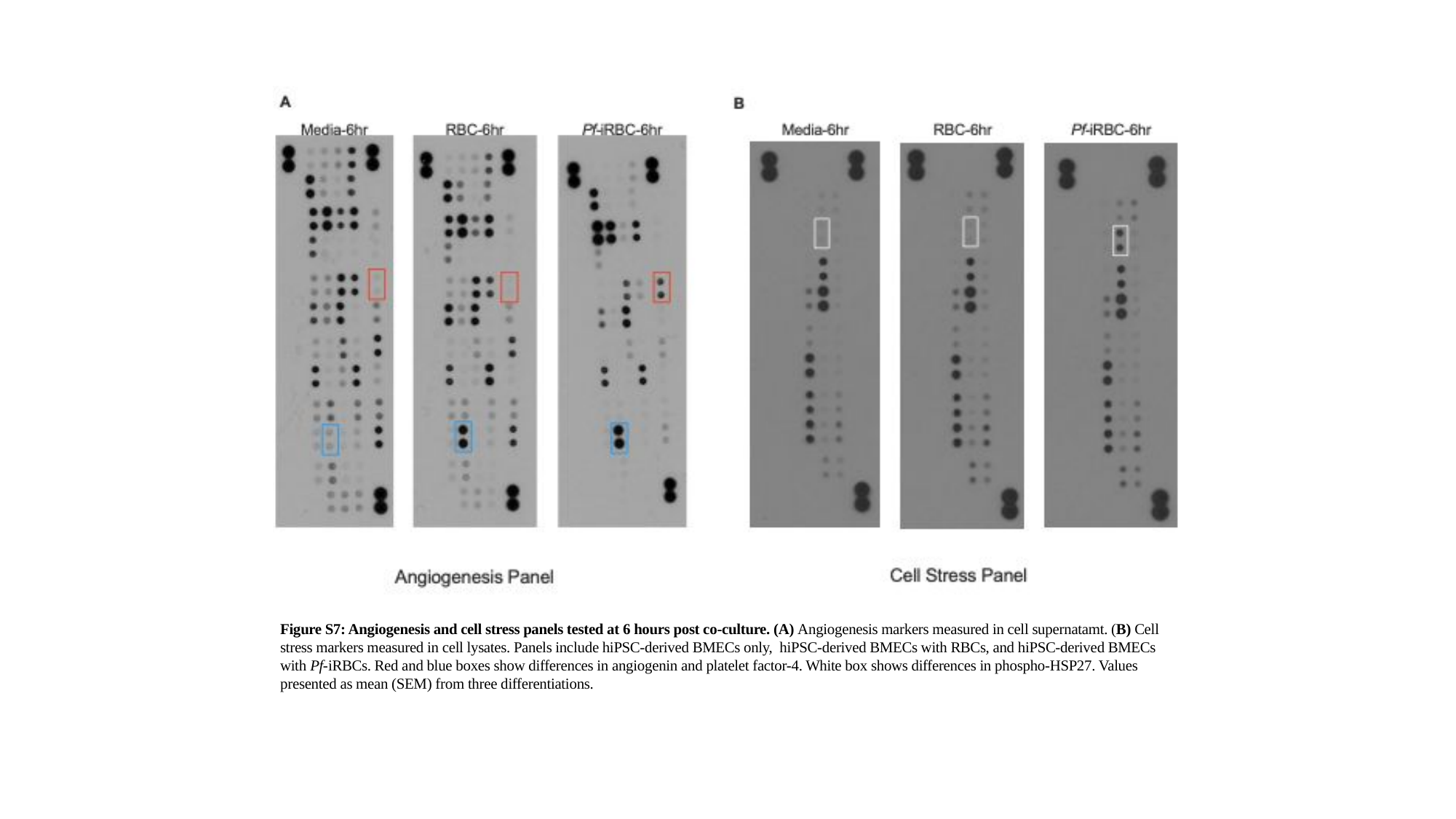

Figure S7: Angiogenesis and cell stress panels tested at 6 hours post co-culture. (A) Angiogenesis markers measured in cell supernatamt. (B) Cell stress markers measured in cell lysates. Panels include hiPSC-derived BMECs only, hiPSC-derived BMECs with RBCs, and hiPSC-derived BMECs with Pf-iRBCs. Red and blue boxes show differences in angiogenin and platelet factor-4. White box shows differences in phospho-HSP27. Values presented as mean (SEM) from three differentiations.
